# Polymorphisms in CISH Gene Are Associated with Persistent Hepatitis B Virus Infection in Han Chinese Population

**DOI:** 10.1371/journal.pone.0100826

**Published:** 2014-06-25

**Authors:** Zhangyong Hu, Jinliang Yang, Yangping Wu, Guolian Xiong, Yali Wang, Jun Yang, Lan Deng

**Affiliations:** 1 State Key Laboratory of Biotherapy and Cancer Center, West China Hospital, West China Medical School, Sichuan University, Chengdu, China; 2 Department of Infection Disease, The First Affiliated Hospital of Chengdu Medical College, Chengdu, Sichuan Province, China; Kliniken der Stadt Köln gGmbH, Germany

## Abstract

**Background and Aim:**

Cytokine-inducible SRC homology 2 domain protein (CISH) is the first member of the suppressors of cytokine signaling (SOCS) protein family. An association between multiple CISH polymorphisms and susceptibility to infectious diseases has been reported. This study aimed to investigate the possible association of these single nucleotide polymorphisms (SNPs) in CISH gene with different outcomes of Hepatitis B virus (HBV) infection.

**Methods:**

1019 unrelated Chinese Han subjects, including 240 persistent asymptomatic HBV carriers, 217 chronic hepatitis B patients, 137 HBV-related liver cirrhosis patients, and 425 cases of spontaneously recovered HBV as controls, were studied. Four SNPs (rs622502, rs2239751, rs414171 and rs6768300) in CISH gene were genotyped with the snapshot technique. Transcriptional activity of the CISH promoter was assayed *in vitro* using the dual-luciferase reporter assay system.

**Results:**

At position rs414171, A allele and AA genotype frequencies were significantly higher in the HBV-resolved group as compared to the persistent HBV infection group. At position rs2239751, TT genotype was further observed in the HBV-resolved group. Using asymptomatic HBV carriers as controls, our results indicated that the rs414171 and rs2239751 polymorphisms were unrelated to HBV progression. The other two SNPs (rs622502 and rs6768300) showed no association with persistent HBV infection. Haplotype analysis revealed that the GGCA haplotype was associated with spontaneous clearance of HBV in this population. Moreover, luciferase activity was significantly higher in the PGL3-Basic-rs414171T construct as compared to the PGL3-Basic-rs414171A construct (p<0.001).

**Conclusion:**

Two SNPs (rs414171 and rs2239751) in the CISH gene were associated with persistent HBV infection in Han Chinese population, but not with HBV progression.

## Introduction

Hepatitis B virus (HBV) infection remains a global health problem. Patients with chronic HBV infection are at risk of developing diverse severe outcomes, including liver cirrhosis, liver failure, or hepatocellular carcinoma (HCC) [1]. Although the precise mechanisms of susceptibility to chronic HBV infection and factors influencing different clinical outcomes are not well understood, accumulating evidence indicates that host factors such as polymorphisms in some key genes could influence the outcomes of patients with HBV infection [2], [3], [4].

The suppressors of cytokine signaling (SOCS) are a family of intracellular proteins, which have been shown to play critical roles in both innate and adaptive immune responses [5], [6], [7], [8]. Cytokine-inducible SH2-containing protein (CISH), the first identified member of the SOCS protein family,can be induced in response to stimulation by different cytokines, and negatively regulates the signaling of cytokines, in particular IL-2. Recent studies have suggested that CISH is involved in human diseases [9], [10], and polymorphisms in CISH gene are associated with susceptibility to infectious diseases [11], including bacteremia, malaria, tuberculosis and Hepatitis B [12]. Therefore, we aimed to investigate the possible relationship between these CISH polymorphisms (rs622502, rs2239751, rs414171 and rs6768300) and the different outcomes of HBV infection in Chinese Han population.

## Materials and Methods

### Study population

The study participants were selected from the first affiliated hospital of Chengdu medical college in Sichuan province of southwestern China. The study protocol adhered to the tenets of the Declaration of Helsinki, and was approved by the ethics committee of the first affiliated hospital of Chengdu medical college. All patients signed an informed consent prior to enrollment in the study. A total of 594 persistent HBV-infected patients of unrelated Chinese Han population were included in this study, of which 240 were chronic asymptomatic HBV carriers (AsC), 217 patients had active chronic hepatitis B (CHB) infection, and 137 were liver cirrhosis (LC) patients. Persistent HBV infection was defined as presence of positive HBsAg for at least 6 months. A group of 425 individuals with spontaneous clearance of HBV (HBV-resolved) served as the control group. Clinical criteria for HBV-resolved group were: negative for HBsAg, plus positive for both anti-HBs and anti-HBc, or plus anti-HBs positive without any history of hepatitis B vaccination. About 90% of HBV-resolved cases were anti-HBc positive in our cohort. All individuals were tested to exclude Hepatitis C (HCV), Hepatitis D (HDV), and human immunodeficiency virus (HIV) infections(The baseline characteristics of the study subjects are summarized in Table 1).

**Table 1:** Baseline characteristics of study subjects.

	HBV-resolved(n = 425)	AsC(n = 240)	CHB(n = 217)	LC(n = 137)
Male	191(44.90%)	147(61.25%)	148(68.20%)	109(79.56%)
Female	234(55.06%)	93(38.75%)	69(31.80%)	28(20.44%)
age	41.08±14.38	36.23±11.98	40.65±13.13	49.69±11.63
HBsAg+	0	240(100.0%)	217(100.0%)	137(100%)
Anti-HBs+	410(96.47%)	0	0	0
HBeAg+	0	56(23.33%)	98(45.16%)	21(15.33%)
Anti-HBc+	398(93.65%)	138(57.50%)	142(65.44%)	107(78.10%)
ALT>40(IU/L)	0	0	201(92.63%)	46(33.58%)

### DNA isolation and genotyping

Genomic DNA was isolated from peripheral whole blood using TIANamp blood DNA kit. The concentration and purity of the DNA were determined with a NanoDrop spectrophotometer, and the DNA was diluted to a final concentration of 10 ng/µL. Multiplex PCR reactions were designed to amplify CISH fragments covering all objective CISH SNP loci(The primers used in this study were shown in Table S1). These PCR reactions were performed in a total volume of 20 µl containing 1×GC Buffer I (Takara), 3.0 mM Mg2+, 0.3 mM dNTP, 1 U of HotStart Taq DNA polymerase (Takara), 2 µl of multiple primers, and 1 µl of genomic DNA. The cycling conditions were 95°C for 2 min, 11 cycles at 94°C for 20 s, 65°C for 40 s, 72°C for 1.5 min; 24 cycles at 94°C for 20 s, 59°C for 30 s, 72°C for 1.5 min, followed by 72°C for 2 min, and finally kept at 4°C. 15 µl of mixed PCR products were then incubated with 5 U of shrimp alkaline phosphatase (SAP) and 5 U of Exonuclease I (ExoI) at 37°C for 1 hour, and then inactivated at 75°C for 15 min. 10 µl of extension reaction contained 5 µl of SNaPshot Multiplex Kit (ABI), 2 µl of purified PCR products, 1 µl of extension primers, and 2 µl of ultrapure water. The ligation cycling programs were 96°C for 1 min; 28 cycles at 96°C for 10 s, 52°C for 5 s, 60°C for 30 s, and then kept at 4°C. 0.5 µl of purified extension products were loaded in ABI 3130XL sequencer (Applied Biosystems, USA), and the primary data were analyzed by GeneMapper 4.1 (Applied Biosystems, USA).

### Luciferase assay with SNP at rs414171 in the CISH promoter

The Dual-Luciferase Reporter Assay System (Promega, USA) was chosen to identify the activity of CISH promoter. All experiments were performed according to the manufacturer's guidelines. Briefly, primer sequences were designed using Primer Premier 5.0 and Oligo software as follows: Sense: 5′ –CTA**GCTAGC**GCTGCCTAATCCTTTGTCTG-3′ (the sequence in bold is an NheI restriction site), Antisense: 5′-CCG**CTCGAG**CACGCCGACAGACCTCCTTG-3′ (the sequence in bold is an XhoI restriction site). Consensus primers were designed to CISH regions, which includes rs414171 A>T polymorphisms were used to amplify a 493 bp sequence from human genomic DNA by polymerase chain reaction (PCR). In this clone, all 493 bp nucleotides were located upstream of the first exon of CISH sequence. DNA fragments corresponding to CISH promoter region from nucleotides −12 to −505 (relative to the first nucleotide of the open reading frame of CISH) were inserted upstream of the firefly luciferase gene in the pGL3-Basic plasmid vector (Promega, USA) in separate steps, and were named as basic AA and basic TT. All reporter constructs were confirmed by direct sequencing. 293T cells were cultured in Dulbecco's modified Eagle's medium (DMEM, Gibco, USA) supplemented with 10% heat-inactivated fetal bovine serum (Biowest, France). For luciferase assays, 293T cells were plated in a 96-well plate and grown to 60% confluency. For transfections, 0.1 µg of a given reporter construct and 10 ng of pRL-TK (renilla, internal control, Promega) per well were co-transfected using Lipofectamine 2000 Reagent (Invitrogen, USA) according to the manufacturer's instructions. After transfection for 48 hours, cells were lysed in 100 µl of 1× passive lysis buffer (PLB buffer from Promega) at room temperature for 20 min. For dual luciferase assay, 30 µl of lysate was aliquoted into a 96-well plate for measuring firefly luciferase (50 µl of LAR II) and renilla luciferase (50 µl of stop and glow buffer) activity. Fluorescence intensity was read on BioTek Synergy 4 multi-mode microplate reader (BioTek Instruments Inc., USA). All experiments were repeated three times in triplicate.

### Statistical analysis

Hardy-Weinberg Equilibrium (HWE) assumptions were independently tested for each polymorphism using x^2^ test. The strength of association between the alleles or genotypes and disease status were calculated using SPSS 18.0 software. The odds ratios (ORs) with their 95% confidence intervals (CIs) were computed on the basis of the binary logistic regression analysis, and adjusted for age and gender. P value <0.05 was considered to be statistically significant. Haplotype frequencies were estimated by using PHASE 2.1.

## Results

### Genotyping of CISH polymorphisms

Four SNPs in CISH gene were genotyped in 594 persistent HBV infection and 425 HBV-resolved controls. The genotypic distributions of CISH polymorphisms (rs622502, rs2239751, rs414171 and rs6768300) were found to be in HWE with an minor allele frequency (MAF) of 1% in the southwestern Chinese Han population (Table 2).

**Table 2:** SNP marker information for CISH gene for all subjects studied (persistent HBV infection and HBV-resolved).

Gene	Chr.No	SNPs	position	location	ObesHET	PredHET	HWpval	MAF	Alleles
		rs622502	+3415	intron	0.11	0.11	0.33	0.06	G:C
CISH	3	rs2239751	+1320	exon	0.47	0.47	0.81	0.39	T:G
		rs6768300	−163	promoter	0.12	0.11	0.89	0.06	C:G
		rs414171	−292	promoter	0.48	0.49	0.50	0.44	T:A

### Comparison of CISH gene polymorphisms between HBV-persistent and HBV-resolved groups

The CISH gene and genotype frequencies are presented in Table 3. The alleles and genotype frequencies for SNPs of rs622502 and rs6768300, did not significantly differ between persistent HBV infection and HBV-resolved groups (all p>0.05). Regression analyses showed no association between the above-mentioned SNPs and different outcomes following HBV infection in this study (data not shown). While rs414171 showed significant association with persistent HBV infection, in the case of rs414171 with AA as reference, the TT carriage had a significantly higher risk for persistent HBV infection group after adjustments for age and gender (OR = 1.71; CI: 1.19–2.47; x^2^-value = 8.40, p = 0.004). Taking A allele as conference, the OR of T allele carriage for persistent HBV infection was 1.27 (p<0.01). Compared to individuals carrying the GG genotype and G allele of rs2239751, those with TT genotype and T allele had an increased risk of persistent HBV infection with an adjusted OR of 1.63 (95% CI = 1.10–2.37) and 1.23 (95% CI = 1.02–1.47), respectively.

**Table 3:** CISH genotype and allelic frequencies for association with persistent HBV infection in Chinese Han population.

SNPs	Case (n = 594)	Control(n = 425)	OR(95%CI)	x^2^	adjusted p value
rs2239751					
GG	78 (13.13%)	75 (17.65%)	1	6.01	0.05
GT	277 (46.63%)	201 (47.29%)	1.39 (0.95–2.02)	2.92	0.09
TT	239 (40.24%)	149 (35.06%)	1.63(1.10–2.39)	5.99	0.01
G	433(36.45%)	351(41.29%)	1		
T	755(63.55%)	499(58.70%)	1.23(1.02–1.47)	4.92	0.03
rs414171					
AA	107(18.01%)	101(23.76%)	1	8.40	0.02
TA	283(47.64%)	204(48.00%)	1.39(0.99–1.99)	3.67	0.06
TT	204(34.34%)	120(28.24%)	1.71(1.19–2.47)	8.40	0.004
Allele A	497(41.83%)	406(47.76%)	1		
Allele T	691(58.16%)	444(52.24%	1.27(1.07–1.52)	7.06	0.01
rs622502					
GG	529(89.06%)	372(87.53%)	1	1.13	0.57
GC	62 (10.43%)	50 (11.76%)	1.30(0.242–6.70)	0.09	0.76
CC	3 (0.51%)	3 (0.71%)	1.58(0.31–8.17)	0.30	0.59
G	1120(94.28%)	794(93.41%)	1		
C	68(5.72%)	56(6.59%)	1.16(0.81–1.67)	0.65	0.42
rs6768300					
CC	532(89.56%)	373(87.76%)	1	2.32	0.31
GC	61 (10.27%)	49 (11.53%)	0.82 (0.55–1.24)	0.86	0.35
GG	1 (0.17%)	3 (0.71%)	0.24 (0.02–2.37)	1.51	0.22
C	1125(94.70%)	795(93.53%)	1		
G	63(5.30%)	55(6.47%)	1.24(0.85–1.79)	1.24	0.27

Data were calculated by binary logistic regression analysis.

### Association between CISH gene polymorphisms (rs2239751 and rs414171) in different persistent HBV infection groups

To determine whether rs414171 and rs2239751 polymorphisms were associated with different outcomes of persistent HBV infection, a similar analysis was done by comparing case CHB+LC with AsC group, and case LC with AsC group. However, no significant association was observed in any of these comparisons (Tables 4 and 5).

**Table 4:** Genotype and allelic distribution for CISH gene polymorphisms when AsC group was compared to CHB+LC group.

SNP	AsC (N = 240)	CHB+LC (N = 354)	OR(95%CI)	x^2^	adjusted P value
rs2239751					
GG	37(15.42%)	41(11.58%)	1	2.06	0.36
GT	102(42.50%)	175(49.44%)	1.47(0.86–2.51)	2.01	0.16
TT	101(42.08%)	138 (38.98%)	1.29 (0.75–2.22)	0.86	2.51
G	176(36.67%)	257(36.30%)	1		
T	304(63.33%)	451(63.70%)	0.98(0.774–1.25)	0.02	0.90
rs414171					
AA	50(20.83%)	57 (16.10%)	1	2.32	0.31
AT	104(43.33%)	179 (50.56%)	1.45 (1.90–2.33)	2.32	0.13
TT	86(35.83%)	118 (33.33%)	1.31(0.80–2.16)	1.13	0.30
A	204(42.50%)	293(41.38%)	1		
T	276(57.50%)	415(58.62%)	0.96(0.76–1.21)	0.15	0.70

**Table 5:** Genotype and allelic distribution for CISH gene polymorphisms when AsC group was compared to LC group.

SNP	AsC(N = 240)	LC (N = 137)	OR(95%CI)	x^2^	adjusted p value
rs2239751					
GG	37(15.42%)	16(11.68%)	1	3.81	0.15
GT	102(42.50%)	76(55.47%)	1.64(0.76–3.52)	0.39	0.21
TT	101(42.08%)	45(32.85%)	0.99 (0.45–2.20)	0.00	0.99
G	176(36.67%)	108(39.42%)	1		
T	304(63.33%)	166(60.58%)	1.12(0.83–1.53)	0.56	0.45
rs414171					
AA	50(20.83%)	21(15.33%)	1	5.90	0.05
AT	104(43.33%)	80(58.39%)	1.92(0.96–3.85)	3.41	0.07
TT	86(35.83%)	36(26.28%)	1.05(0.50–2.22)	0.02	0.90
A	204(42.50%)	122(44.53%)	1		
T	276(57.50%)	152(55.47%)	1.09(0.81–1.47)	0.29	0.60

### Haplotype analysis

We constructed haplotypes of these four SNPs (rs622502, rs2239751, rs6768300 and rs414171) for association analysis correlating with persistent HBV infection. As compared to the most common GTCT haplotype, only GGCA haplotype was significantly associated with lower risk of chronic HBV infection (OR = 0.79; 95% CI: 0.65–0.95; x^2^ = 6.51, p = 0.003) (Table 6).

**Table 6:** Results of the association test for four SNPs (rs622502, rs2239751, rs6768300 and rs414171) haplotypes in Han population from southwestern China.

Haplotype	Cases(Freq)	Controls(Freq)	OR(95%CI)	chip-square	Adjusted p value
**GTCT**	689(58.00%)	443(52.10%)	1		
GGCA	428(36.03%)	350(41.20%)	0.79(0.65–0.95)	6.51	0.003
CTGA	63(5.30%)	54(6.35%)	0.75(0.51–1.10)	2.18	0.14

The most common haplotype as the reference.

### Effects of rs414171 on transcriptional activity of CISH gene

**Figure 1:**
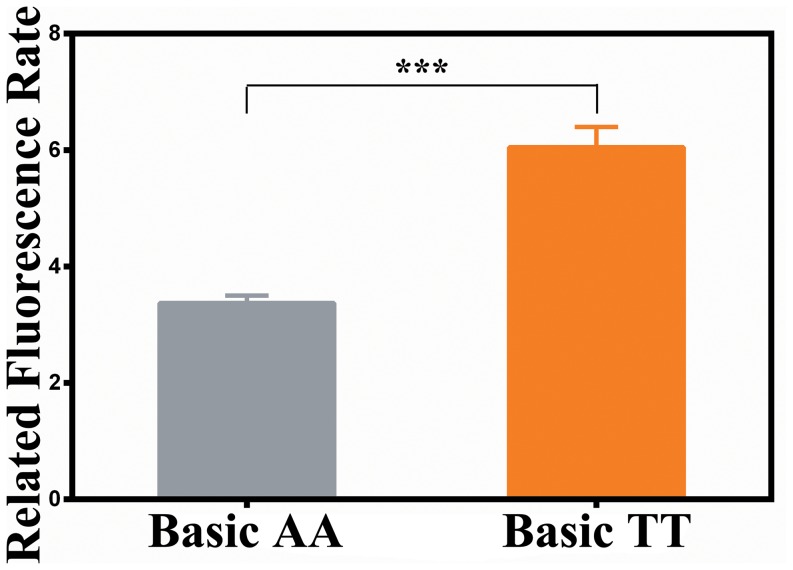
Effect of the rs414171 polymorphism on promoter transcriptional activity. Significantly higher luciferase activities were generated by the pGL3-Basic-T construct as compared to the pGL3-Basic-A construct. (***P<0.001).

To confirm the effects of rs414171 SNP on CISH gene transcription, we analyzed the transcriptional activity of its promoter with a dual-luciferase reporter assay system, and compared the activities of rs414171A and rs414171T alleles using a transient transfection assay in 293T cells. As shown in Figure 1, the pGL3-Basic-rs414171TT reporter showed significantly higher luciferase activity as compared to pGL3-Basic-rs414171AA, suggesting that the A to T polymorphism at position rs414171 may increase the activity of the CISH promoter.

## Discussion

Several methods exist for SNP detection. In the present study, we adapted the snapshot method for SNP detection. As compared to the direct sequencing method, the snapshot method is both time- and cost-efficient, and is therefore, suitable for examining large samples and multilocus genotyping. The accuracy of the assay was validated by direct sequencing [13].

In this study, we investigated four SNPs in the CISH gene, and the correlation of these polymorphisms with HBV clearance and disease progression. The frequency of allele A and AA genotype at position rs414171, and GG genotype at position rs2239751 of CISH gene were higher in the HBV clearance group as compared to the persistence group. This finding is consistent with a recent study conducted by Tong et al. [12]. Individuals carrying the GGCA haplotype are at a higher risk of developing persistent HBV infection as compared to the most common haplotype, GTCT. However, there is no correlation of these four SNPs with disease progression. Our study suggests that the rs414171 and rs2239751 polymorphisms could be associated with the persistence or chronicity of HBV infection, but perhaps they did not correlate with the development of CHB after adjustments for age and gender. Additionally, our findings indicate that SNP rs414171 (A/T) affects the activity of the CISH promoter, and either the A allele or the T allele can substantially drive reporter gene expression. Moreover, significantly higher luciferase activity was found for the rs414171 TT genotype as compared to the AA genotype. This finding suggests that SNP rs414171 is functional, and the substitution of a T for an A can enhance CISH promoter transcriptional activity. However, our results are inconsistent with the previous study [11] in that higher level of CISH gene expression in human PBMCs following Interleukin-2 stimulation is associated with the rs414171AA genotype.

Previous studies have demonstrated that CISH is an immediate-early gene that is induced by several cytokines and T cell receptor (TCR) stimulation. As a result, elevated CISH can negatively regulate cytokine receptor signaling through JAK/STAT pathway, and promote T cell proliferation and survival in a STAT5-independent manner [14], [15]. Since CISH has a pivotal role in immune responses, and it can inhibit the activation of STAT3, STAT5 and STAT6 in T cells, CISH-deficient T cells tend to differentiate into Th2 and Th9 subsets. Also, airway inflammation arises spontaneously in CISH-deficient mice [16], indicating that CISH could be involved in diseases by regulating the differentiation of T cells. By binding to protein kinase Cφ, CISH could enhance FoxP3 expression of natural regulatory CD4^+^ T cells (Tregs), and mediate expansion of Tregs [17], [18], [19], which have been demonstrated to be enhanced in patients with CHB. The expansion of Treg cells in patients carrying rs414171 TT genotype could play a crucial role in persistent HBV infection by modulating HBV-specific immune responses [20], [21], [22].

Strong HBV-specific cytotoxic T cell (CTL) responses were assumed to be the central mechanism in HBV clearance and liver damage during HBV infection, and deficient Th1 immunity accompanied by inefficient CTL responses were found in patients with chronic HBV infection or therapeutic failure [23], [24], [25], [26], [27], [28]. IL-2 is an important Th1 cytokine for inducing polyclonality and multispecificity of the CTL responses following HBV infection. By blocking IL-2/JAK/STAT signal pathway,significantly higher production of CISH could remarkably decrease STAT5 signaling and depress the function of CD8^+^ T cells, which could possibly explain why a patient bearing the rs41417 TT genotype has a greater risk for persistent HBV infection.

In conclusion, this study suggests that the polymorphisms of rs414171 and rs2239751 in CISH gene could possibly be linked to persistent HBV infection in Chinese HBV patients. However, the role of CISH in T cell biology remains unclear with some contradictory findings [29], [30]. Further studies are needed to validate our results and clarify the potential functions of human CISH in HBV infection.

## Supporting Information

Table S1 **Primer sequences used for amplification of CISH gene.**
(DOC)
