# Supplementary material for: Polymorphisms in CISH Gene Are Associated with Persistent Hepatitis B Virus Infection in Han Chinese Population
Source: PLoS One. 2014 Jun 25;9(6):e100826. doi: 10.1371/journal.pone.0100826 (PMC4071005; doi:10.1371/journal.pone.0100826)
Supplement: Table S1 — Primer sequences used for amplification of CISH gene. (DOC) [file pone.0100826.s001.doc]

**Table S1 The information of primer sequences for genotyping the polymorphisms in CISH gene by using snapshot technique**

| Target name Primer sequence |
| --- |
| rs622502F TCCAGCTTCACGCTTCCCTAGA  rs622502R GCTGGACCCAGAGGAGGATCTG  rs622502SF TTTTTTTTTTTTTTTTTTTTTTTTTTTTTTTTTGAAAAGGCCTGCCTCCCCC  rs2239751F AGCCAGTCAGGCTGGAACCTCT  rs2239751R AGGTGAGCACCCCCTTGTAAGC  rs2239751SF TTTTTTTTTTTTTTTTTTTTTTTTTTTTTTTTTTTTTCTAGGTACATGTGTGTGCCCGCT  rs414171F2 CAATCGCGACGCTGAAGGTG  rs414171R TCTCTCCGACCTGCCCCTCT  rs6768300F TTCCTAGAACCGCGGGCTGA  rs6768300R2 TCAAAGTATTTCCTTTCACTGCTCAGG |
